# AI-driven cardiovascular risk prediction in patients with diabetes: bridging algorithmic innovation to equitable clinical application

**DOI:** 10.3389/fmed.2026.1831220

**Published:** 2026-06-02

**Authors:** Hongxuan Li, Zheyi Xu, Yanhui Cen, Xin Liu

**Affiliations:** 1 Guangxi University of Chinese Medicine, Nanning, Guangxi, China; 2 Faculty of Chinese Medicine Science, Guangxi University of Chinese Medicine, Nanning, Guangxi, China

**Keywords:** cardiovascular disease, lipid peroxidation, machine learning, population fairness, prediction model, risk of bias, type 2 diabetes

## Abstract

Machine learning models hold promise to revolutionize cardiovascular disease (CVD) prediction in patients with type 2 diabetes, with algorithms such as neural networks demonstrating superior discriminative performance in internal validations. However, a systematic review has revealed that existing models generally carry a high risk of bias and exhibit poor adherence to transparent reporting standards, severely hindering their clinical translation and real-world application. Furthermore, current models are predominantly developed using populations from Europe and North America, resulting in a critical lack of representativeness for Asian populations, where the burden of cardiovascular disease is particularly heavy. This article argues that the field is undergoing a pivotal transition—from an exclusive focus on algorithmic performance to ensuring clinical equity and fairness. Future advancements should prioritize external validation, calibration-aware assessment, subgroup-specific performance reporting, and cautious integration of biologically plausible biomarkers rather than relying on discrimination alone. Only through this approach can machine learning-driven predictive tools truly bridge the gap between innovation and equitable clinical implementation, ultimately alleviating the global burden of diabetes-related cardiovascular complications.

## Introduction

The global burden of diabetes-related cardiovascular disease is escalating at an unprecedented rate (1, 2). According to the 11th edition of the IDF Diabetes Atlas, approximately 589 million adults aged 20–79 years were living with diabetes worldwide in 2024, and this number is projected to rise further in coming decades (1). Cardiovascular complications—including coronary heart disease, stroke, and peripheral vascular disease—substantially elevate patients' mortality risk, particularly in low- and middle-income countries (2). The World Health Organization reports that approximately 80% of global cardiovascular disease deaths occur in low- and middle-income countries (2). Diabetes-related health expenditure has also risen markedly, reaching at least USD 1 trillion globally in 2024 (1).

Against this backdrop, early identification of high-risk individuals and implementation of targeted interventions have become central priorities in public health. Traditional risk assessment tools, such as the ACC/AHA Pooled Cohort Equations (PCE) and the Framingham Risk Score, remain widely used in clinical practice but suffer from notable limitations in patients with diabetes (3). Although these tools are clinically familiar and easy to apply, their transportability across Asian populations is imperfect, and recent evidence suggests that local recalibration is often necessary to maintain acceptable calibration and clinical usefulness (4).

The advent of machine learning models offers hope for overcoming these challenges (5). Unlike traditional regression models reliant on fixed assumptions and linear frameworks, machine learning can autonomously discern non-linear risk patterns from high-dimensional data, iteratively train and validate across algorithms, and identify optimal performers through comparative evaluation. Numerous studies have demonstrated that algorithms such as neural networks and gradient boosting machines exhibit superior discriminative ability over traditional methods in predicting cardiovascular complications in diabetes (6–8).

Nevertheless, a significant gap persists between technological potential and clinical reality. A systematic review by Kee et al. (6) of machine learning studies highlighted both the strengths of these algorithms and critical shortcomings in the field: widespread risk of bias, low adherence to transparent reporting standards, and severe imbalances in population representativeness. More recent reviews have further confirmed these problems and emphasized the major limitation imposed by inadequate independent external validation, which directly restricts model generalizability and real-world deployment (7, 8).

**Figure 1:**
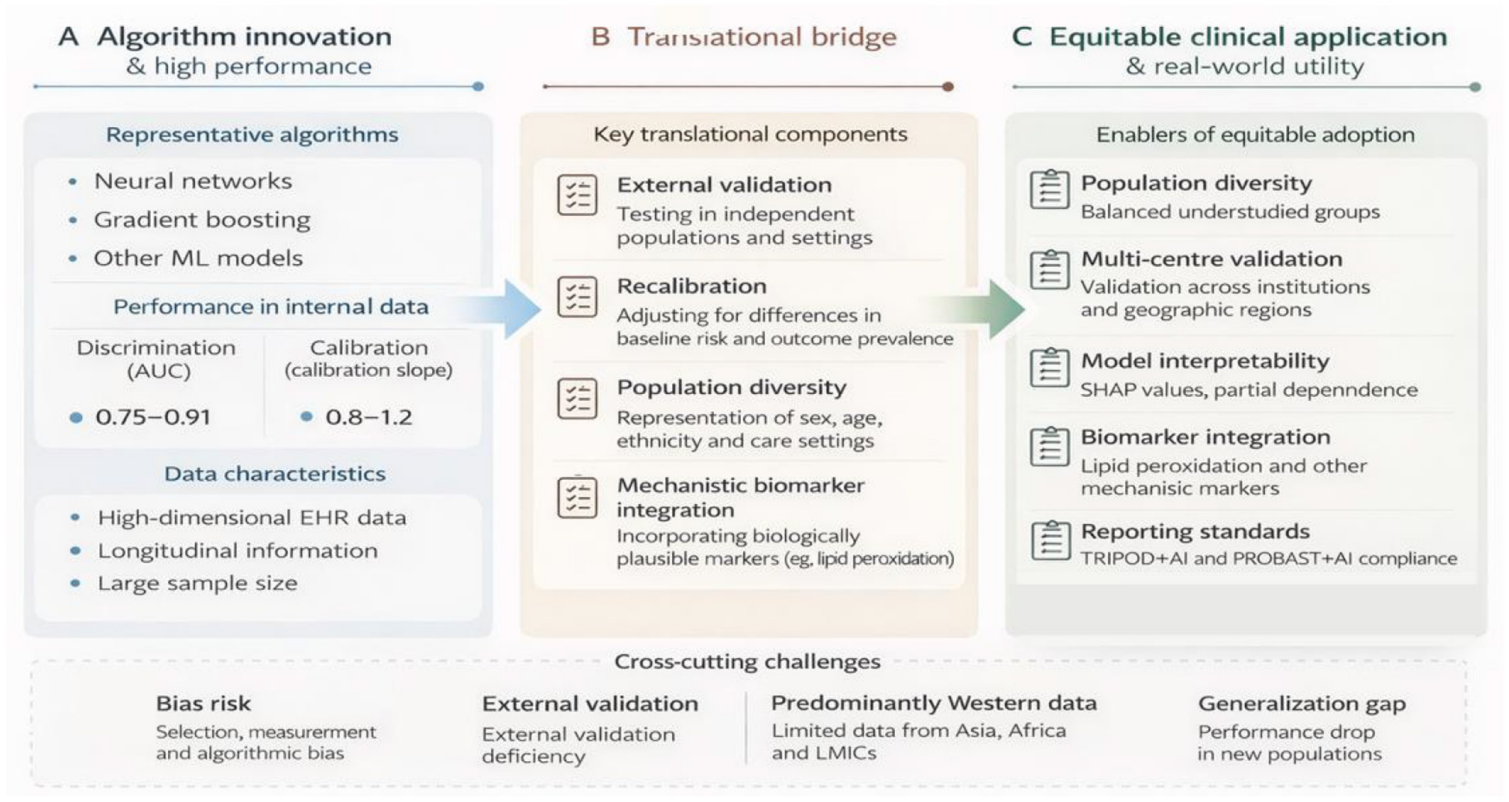
From algorithmic performance to equitable clinical application: a translational framework for machine learning-based cardiovascular risk prediction in patients with type 2 diabetes.

These findings raise a fundamental question: when algorithms demonstrate exceptional performance in published papers, can we be confident in their reliability across diverse real-world populations? This article explores the opportunities and challenges of machine learning in cardiovascular risk prediction for patients with diabetes, advancing a core argument: the future of this field should not be judged by discrimination alone, but by whether models remain transparent, transportable, well-calibrated, and clinically acceptable across relevant subgroups. In this perspective, fairness is therefore treated pragmatically—as the extent to which a model avoids systematically worse performance or miscalibration in underrepresented populations. Recent methodological frameworks, including TRIPOD+AI and PROBAST+AI, further support this shift from performance-centered development toward clinically trustworthy and equitable prediction modeling (9, 10). This transformative process can be intuitively illustrated in Figure 1, which depicts a framework bridging from algorithmic innovation and high performance (left side) to equitable clinical application and real-world utility (right side). Key bridging elements include population diversity, integration of mechanistic biomarkers, adherence to reporting standards, and methodological rigor. In the revised interpretation, these bridging elements are more specifically defined as external validation, local recalibration, subgroup-aware evaluation, and transparent reporting (23, 24).

## Machine learning advantages and methodological concerns in cardiovascular risk prediction

The core strengths of machine learning in cardiovascular risk prediction lie in its capacity to handle complex, high-dimensional data and to adapt effectively to class imbalance, enabling the identification of non-linear risk patterns that traditional models often fail to capture (11). Rather than focusing on isolated “best-case” results, the more consistent message across the literature is that machine learning models can improve discrimination or reclassification in derivation datasets, especially when longitudinal or high-dimensional EHR features are available (8, 11–13). Neural networks and gradient boosting approaches have shown promising performance in several cohorts, and a recent meta-analysis reported a pooled AUC of 0.753 across AI-based cardiovascular prediction models (8). These technical advances provide finer risk stratification for personalized intervention and suggest that machine learning may complement existing diabetes management strategies.

Nevertheless, these impressive performance gains are accompanied by significant methodological concerns. Despite excellent algorithmic results in many studies, systematic evaluations have revealed a high prevalence of risk of bias, primarily stemming from deficiencies in the participant selection domain, outcome measurement domain, and analysis domain (6, 7). A particularly concerning issue is the low adherence to transparent reporting standards: compliance with the TRIPOD checklist averaged only 53.75% across reviewed studies (6). Such reporting deficiencies make it difficult for readers to independently assess model validity, transportability, and clinical relevance. As a consequence, even models that perform exceptionally well on development datasets often show substantially lower or uncertain performance in external settings, highlighting a critical limitation in generalizability (7, 8). Recent guidance now makes clear that clinical prediction studies using machine learning should report not only discrimination, but also calibration, intended use, validation strategy, and risk-of-bias considerations in a structured way (9, 10).

## Global research imbalance and heterogeneity within Asian populations

The development of current diabetes-related cardiovascular risk prediction models starkly reflects imbalances in global research resources and data availability. A large proportion of studies rely on populations from Europe and North America, with inadequate representation of Asian and other non-Western groups, directly compromising clinical applicability in high-burden regions (6, 7). At the same time, recent studies from Malaysia and China suggest that regionally developed machine learning models can improve performance in local cohorts, but these same studies also show why local validation, calibration assessment, and broader external testing remain essential before wider implementation (14, 15).

Furthermore, substantial heterogeneity exists even within Asian populations, adding another layer of complexity to risk prediction. Treating “Asian populations” as a homogeneous entity—or extrapolating models developed in one Asian subgroup to all Asian individuals—can introduce systematic prediction bias. Recent evidence from two population-based multi-ethnic cohorts in Singapore indicates that established cardiovascular risk models can remain useful in Asian populations only after appropriate local recalibration, and calibration performance may still differ across models and subgroups (4). This means that “Asian underrepresentation” is not the only problem; intra-Asian heterogeneity, lack of recalibration, and limited subgroup evaluation are equally important fairness concerns (16, 17).

Taken together, the evidence points to a central conclusion: even within the same geographic region, cardiovascular risk characteristics vary markedly across ethnic and healthcare subgroups. Assuming homogeneity across Asian populations or generalizing models developed from a single subgroup to the broader Asian population risks introducing systematic predictive bias and undermining equitable clinical utility.

## Integration of mechanistic biomarkers

Table 1 summarizes the key evidence addressed in this article and shows that population heterogeneity represents only one dimension of the translational challenge. A deeper bottleneck lies in the fact that many current models remain largely confined to associative prediction—identifying variable combinations correlated with outcomes—without directly engaging the underlying pathophysiological processes of the disease. Among candidate pathways, oxidative stress and lipid peroxidation deserve attention because they are biologically linked to endothelial dysfunction and vascular injury in diabetes; however, this does not yet justify routine inclusion of such biomarkers in all machine learning models (18–21).

**Table 1:** Summary of key evidence on machine learning for cardiovascular risk prediction in patients with type 2 diabetes.

Domain	Main evidence	Key data	Main limitation
Model performance	ML models often outperform conventional scores in derivation cohorts	AUCs around 0.75–0.91 have been reported across reviews and cohort studies (8, 12–15)	Mostly internal or limited validation
External validation	Independent validation remains insufficient	Replicability and transportability remain weak in many published studies (7, 8)	Clinical deployment is premature without broader validation
Population representativeness	Western cohorts remain overrepresented	Asian populations are underrepresented, and Asian subgroups are heterogeneous (4, 6, 7, 14–16)	Risk of miscalibration after transport
Reporting quality	Transparency remains suboptimal	Mean TRIPOD adherence 53.75% in reviewed studies (6)	Reproducibility and appraisal concerns
Biomarker integration	Oxidative stress and lipid peroxidation are biologically relevant	Mechanistic association is plausible, but comparative predictive evidence remains limited (18–22)	Incremental value and standardization remain unclear

The association between lipid peroxidation and diabetic cardiovascular complications has been extensively discussed in the literature (18, 19, 21, 22). Oxidative stress plays a central role in the shared pathophysiology of diabetes and cardiovascular disease, with lipid peroxidation representing one of its major downstream consequences. Studies have shown that oxidized lipid products and related oxidative stress markers may reflect vascular injury, inflammatory activity, and cardiometabolic dysfunction (18–22). This supports the idea that lipid peroxidation markers may serve as mechanistically plausible candidate predictors; however, the present evidence base remains heterogeneous in marker selection, assay platform, and standardization, and direct comparative evidence against standard clinic-based models is still limited (20–22).

Nevertheless, incorporating lipid peroxidation biomarkers also presents practical challenges. First, measurement of these biomarkers requires standardization to ensure comparability across studies. Second, the addition of laboratory assays may increase the financial and logistical burden on healthcare systems in low- and middle-income countries, potentially limiting implementation. Future research must strike a balance between predictive accuracy and real-world feasibility, exploring tiered strategies tailored to resource availability. In resource-rich settings, inclusion of lipid peroxidation biomarkers may improve biological relevance; in resource-constrained environments, priority should be given to robust collection of basic clinical variables, external validation, and local recalibration. More importantly, future studies should quantify whether these biomarkers improve discrimination, calibration, and net clinical benefit beyond baseline clinical models instead of inferring translational value from biological plausibility alone (9, 10, 20, 22).

## Future directions and opportunities for innovation

Future research should prioritize multicenter studies in high-burden regions such as Southeast Asia and South Asia to develop more locally adaptive prediction models for diabetic cardiovascular complications. Recent studies from Malaysia and China illustrate both the promise of regionally tailored machine learning approaches and the continuing need for broader external validation (14, 15). Evidence from Singapore demonstrates that recalibration using local data can substantially enhance model performance and improve calibration in multi-ethnic Asian populations (4). At the same time, adherence to TRIPOD+AI and PROBAST+AI should be treated as a methodological expectation, not an optional add-on, because transparency and structured bias appraisal are necessary for evaluating whether apparently strong models are actually fit for clinical use (9, 10).

Moreover, integrating mechanistic biomarkers—such as those related to lipid peroxidation—holds potential to improve biological relevance and possibly predictive performance (18–22). Future studies should rigorously quantify how the addition of such biomarkers affects discrimination, calibration, and transportability, particularly across diverse populations. Model development processes must also emphasize fairness by ensuring diversity in data collection and by stratifying performance reporting according to population characteristics during validation and deployment phases. This is especially important because a recent scoping review found that fairness metrics are rarely reported in clinical risk prediction studies, while a recent cardiovascular AI perspective emphasized that bias can arise throughout problem formulation, dataset construction, model development, validation, and implementation (16, 17). Where feasible, model comparison should therefore extend beyond AUC to include calibration, subgroup error distribution, and decision-curve-oriented evaluation of clinical usefulness (9, 10, 16, 17).

## Conclusion

Machine learning models hold immense promise for the prediction of diabetes-related cardiovascular disease, yet their clinical translation continues to face substantial hurdles. While these models often exhibit excellent performance in controlled or internally validated settings, issues of data bias and limited external generalizability restrict their widespread adoption, particularly in high-risk regions such as Asia. The predominance of models developed from European and North American populations results in inadequate representativeness for other groups, potentially exacerbating healthcare disparities. The central challenge is therefore no longer whether machine learning can improve discrimination, but whether it can do so in a way that is transportable, transparent, calibrated, and clinically equitable. The future trajectory of this field must shift from an exclusive focus on algorithmic accuracy toward ensuring model fairness, interpretability, and broad clinical applicability. By incorporating emerging mechanistic biomarkers and addressing population-specific differences, machine learning-driven tools can deliver more personalized and equitable risk assessment, thereby advancing the realization of precision medicine. However, biomarker integration should remain evidence-led rather than speculative, and claims of readiness for clinical use should be supported by external validation, subgroup assessment, and calibration-aware evaluation. Ultimately, prioritizing fairness, biological plausibility, and real-world utility will be essential to drive meaningful progress in this domain.

## Data Availability

The original contributions presented in the study are included in the article/supplementary material, further inquiries can be directed to the corresponding author.
